# Case of Hermaphroditism

**Published:** 1851-11

**Authors:** John Neill


ARTICLE III.

*Case of Hermaphroditism.* Dr. JNO. NEILL communicated to the College of Physicians the following curious example of Hermaphroditism, in a black brought to the anatomical rooms of the University of Pennsylvania.

She dressed as a female, and was apparently twenty-five or thirty years of age, judging by her teeth and general appearance. Very little information could be obtained concerning her habits and propensities. She resided among the degraded blacks in the lower portion of the city, and died from drunkenness and exposure, according to the verdict of the coroner's jury.

From a superficial view of the pelvis and genitals, almost any one would have pronounced the subject to have been a *hypospadic male*, notwithstanding the large mammæ and the want of hair upon the face.

The breadth of the shoulders compared with the narrowness of the hips, and the form and development of the limbs would, alone considered, have indicated the male sex.

The representative penis was five inches in length, and one inch in diameter; and the skin, prepuce, glans, corona, fossa navicularis, and orifice of the urethra presented an appearance like that of a penis. But, by lifting up or turning aside the penis, it was found that the fossa navicularis was split, and that the urethra was wanting. In the place of the urethra there was a groove reaching from the glans penis to an oblique opening in the perineum. The cuticle lining the groove was thin and shining; it was also deficient in pigmentary cells. On each side of this groove there was a fold of skin commencing near the middle of the side of the penis, and stretching around the perineal orifice. The interior of this fold showed it to be the analogue of the nymphæ or corpus spongiosum.

The perineal opening was the commencement of a passage common to the bladder and vagina, and its diameter was equal to that of a common-sized catheter, although the orifice appeared much larger, owing to its obliquity.

The scrotum existed upon one side only. It was corrugated with transverse rugæ, and covered as usual with hairs and sebaceous follicles. To the touch it gave the idea that it contained two hard bodies.

*Internal Organs.*—These were completely female, though not perfectly developed. The dissection was commenced by opening the abdominal cavity, and the contents of the pelvis were examined in connection with the external parts.

The bladder was natural in position and size. There was no prostate, and the urethra was about one inch in length, and opened into the perineal passage.

The uterus was small, but symmetrical; to its sides were attached the broad ligaments, holding it in its proper relation to the rectum and bladder.

The Fallopian tube of the right side had no free and fimbriated extremity, be terminated in a sac which was adherent to the ovary.

The ovaries were small, spherical, and corrugated; a section exhibited the usual filbrous tissue and visicles.

The right round ligament of the userus was exceedingly thick, and appeared to be muscular; but, upon examination with the microscope, it was found to be composed of white and fibrous tissue. It reached to the bottom of the scrotum, where it was firmly attached.

The *scrotum* contained an irreducible omental hernia, probably congenital. The hernial sac contained also a small hardened mass, which was supposed to be a representative testicle, but it contained no true glandular structure or excretory tube. The vagina was of the proper length, but extremely narrow, especially where it approached the perineal orifice.

The above case would be classified under the head of

"*spurious heamaphroditism*" in the female, according to Professor Simpson's article on this subject in the *Cyclopædia of Anatomy and Physiology.—Quarterly Summary of Translations.*

